# Impact of the Global Budget Payment System on Expenditure of Cardiovascular Diseases: An Interrupted Time Series Analysis in Shanghai, China

**DOI:** 10.3390/ijerph16081385

**Published:** 2019-04-17

**Authors:** Guanshen Dou, Yilin Zhang, Yunzhen He, Qiaoyun Huang, Yingfeng Ye, Xinyu Zhang, Weibing Wang, Xiaohua Ying

**Affiliations:** 1 NHC Key Laboratory of Health Technology Assessment (Fudan University), Department of Health Economics, School of Public Health, Fudan University, Shanghai 200032, China; gsdou14@fudan.edu.cn (G.D.); 16211020031@fudan.edu.cn (Y.H.); 17111020031@fudan.edu.cn (Y.Y.); 13301020126@fudan.edu.cn (X.Z.); 2 London School of Economics and Political Science, Houghton Street, London WC2A 2AE, UK; yilinzh2008@gmail.com; 3 School of Public Health, Kunming Medical University, Kunming 650500, China; huangqiaoyun821@163.com; 4 Department of Epidemiology, School of Public Health, Fudan University, Shanghai 200032, China; wwb@fudan.edu.cn

**Keywords:** global budget payment system, cardiovascular diseases, medical expenditure, China

## Abstract

Since few studies evaluated the impact of the global budget payment system (GBPS) over time, and by expenditure type, this paper aims to evaluate the impact of the GBPS on expenditure of inpatients, and explores how hospitals curb the expenditure in patients with cardiovascular diseases (CVDs) in Shanghai. We built a time series model with the monthly expenditure of CVDs from 2009 to 2012. We evaluated the instant impact and trends impact of the GBPS and analyzed results based on medical expenditure types (e.g., drug, examination, cure, unclassified items), discharge number, and expenditure per capita. We found GBPS instantly dropped the medical expenditure by Chinese Yuan (CNY) 55.71 million (*p* < 0.001), and decreased the monthly increasing trend by CNY 4.23 million (*p* = 0.011). The discharge number had 10.4% instant reduction and 225.55 monthly decrease (*p* = 0.021) while the expenditure per capita experienced fewer changes. Moreover, the expenditure of drug and cure had an instant reduction of CNY 28.31 million and 16.28 million (*p* < 0.001). In conclusion, we considered the GBPS is an effective solution to control the expenditure of CVDs by decreasing the discharge number, and a focus on the drug and cure expenditures lead to greater spend reduction than other types of expenditures.

## 1. Introduction

Controlling the increasing health expenditure is one of China’s greatest challenges. Prior to 2011, the conventional payment system for healthcare provider reimbursement was fee-for-service (FFS). The FFS model promotes the provision of more services and consequently induces greater medical expenditure [1]. In contrast, the global budget payment system (GBPS) controls for increasing expenditure, under which provider organizations are provided a prospective reimbursement budget based on their patient population. This has been considered as one of the most promising approaches to control increasing health expenditure [2,3]. Research from the United States has demonstrated the GBPS’ effectiveness for controlling health expenditure [4].

Shanghai, one of the richest cities in China, in 2009 had a total health expenditure per capital of Chinese Yuan (CNY) 3962.76, more than two times the national average [5,6]. That same year, Shanghai launched the GBPS to control for growing medical expenditure. As part of the first year launch, three tertiary hospitals were selected to pilot the GBPS. In the second year, 2010, the GBPS was piloted in ten additional tertiary hospitals and all secondary hospitals. In its last implementation phase in 2011, the GBPS was introduced to all remaining tertiary hospitals.

The implementation of the GBPS in Shanghai was comprised of several considerations, including budget determination, budget adjustment, and annual accounts. The Human Resources and Social Security Bureau (HRSSB) determines each hospital’s annual budget every year via negations with representatives of hospitals. The budget of each hospital is built on service volume, service quality, and a prospective increasing rate.

The HRSSB reimburses claims to the hospital directly each month, and the amount is capped in accordance to the annual budget. At the end of year, any remaining funds are distributed back to the HRSSB and hospitals. Where hospitals overspend, the hospital is responsible for paying part of the extra expenditure. For example, a tertiary hospital had to pay 60 percent of the extra expenditure itself in 2012.

In this study, we focus on expenditure with respect to cardiovascular diseases (CVDs). CVDs, which include hypertension and ischemic heart diseases, represent some of the most serious diseases among the Chinese population. For example, hypertension prevalence rate was 44.7% for people over 55 years in China over a three-year study from 2014 to 2017 [7]. Consequently, CVDs have caused substantial increases in medical expenditure every year, which places additional financial pressure on the Urban Employee’s Basic Medical Insurance (UEBMI). The UEBMI, one of the basic types of insurance in China, is the largest medical service payer in the country and covers more than 200 million employers [5]. In 2012, the UEBMI covered 15.9 million lives in Shanghai. The UEBMI covers some fees and the insured pays out-of-pocket (OOP) any remaining fees that are not covered by the UEBMI. 

While related studies have shown the positive impact of the GBPS on limiting unnecessary medical utilization and managing medical expenditure, there is scarcity in studies that evaluate the impact of the GBPS over time, and by expenditure type [8,9,10,11,12]. In addition, the effectiveness varies by scenario among regions, so it is necessary to evaluate the impact of GBPS in Shanghai. In this paper, we aimed to evaluate the GBPS’ impact on the medical expenditure of inpatients diagnosed with CVDs, and we further evaluated the variation in GBPS’ impact by type of expenditure.

## 2. Materials and Methods 

### 2.1. Study Population

The study population consists of all UEBMI insured hospitalizations as a result of CVDs in Shanghai from 2009 to 2012. The CVDs were categorized by the International Classification of Diseases Revision 10 (ICD 10) codes (I00-I99), which mainly contained hypertensive diseases (I10-I15) and ischemic heart diseases (I20-I25). The inpatient sample originated from all hospitals in Shanghai, which included 767 primary hospitals, 221 secondary hospitals, and 73 tertiary hospitals. The inpatient sample included 1,124,356 hospitalizations, which account for more than 20% of all UEBMI insured hospitalizations in Shanghai during the study period.

### 2.2. Data Collection and Measures

The daily medical expenditure data is based on inpatients hospitalized as a result of a CVD from 2009 to 2012. These inpatients were also Shanghai residents covered by UEBMI. We totaled the daily data by month and analyzed monthly expenditure. We defined the study period as 2009 to 2012 to avoid potential interference from the public hospital reform in Shanghai, which began at the end of 2012. From January 2009 to March 2010, we established a foundational dataset to align with the UEBMI fund annual closing in March. In April 2010, the GBPS pilot formally began, and the annual study periods ran from April 2010 to March 2011, and from April 2011 to December 2012. 

Each of hospitalization was recorded by each contracted hospital and sent to HRSSB by internal network. The HRSSB summed the total expenditure by day and delivered the preprocessed data to the research team. The numbers of CVD hospitalizations were aggregated by gender and age, and the expenditure was aggregated by service. Prior to analysis, patient records were de-identified and only the total daily costs of respiratory diseases hospitalizations were assessed. Authors had no right to review individual patient’s information and could not come in contact with any patient during research. Consequently, the Ethics Committee of the School of Public Health, Fudan University, granted the study an exemption from ethical review.

### 2.3. Outcomes and Variables

As the GBPS was set to control excessive increasing medical expenditure, the main outcome indicator we assessed to reflect the GBPS’ effect was the monthly medical expenditure. And, to assess if the GBPS could relieve the economic burden on patients, we included two additional outcome indicators: the discharge number and expenditure per capita. Finally, the expenditure was divided by characteristics of each service into four main categories: drug, examination, cure, and unclassified items expenditures (mainly included the expenditure of medical supplies, transfusion, and oxygen therapy). These classifications allowed us to evaluate for the types of expenditure most sensitive to the GBPS.

The key variables used to evaluate the GBPS impact by times series analysis were the instant change and the monthly trend change of the outcomes, after the interrupt points. In our study, we set two interrupt points to represent the GBPS pilot and the GBPS full implementation. We adopted two binary variables to divide the baseline group, the GBPS pilot group, and the GBPS full implementation group. Two continuous variables, beginning from each time point, were adopted to reflect the monthly trend change of outcomes, after the policy points. Another continuous variable was used to reflect the linear time trend to control for omitted trending variables, such as progress in medicine and treatment; we separated these variables so that we could control for the impact of the GBPS.

Patient characteristics and seasonal factors were included as covariance. Research has shown that age and gender are associated with medical expenditure in some diseases [13,14,15]; consequently, we selected these two characteristics. With respect to age, we looked at the proportion of patients older than 65. With respect to gender, we looked at the proportion of male patients. Additionally, seasonal factors have demonstrated significant impact on morbidity and mortality in respiratory diseases [16,17,18,19]. In our study, we divided one year into four quarters and designated the months of January, February and March as the first quarter. 

### 2.4. Statistical Analysis

An interrupted time-series analysis (ITS) with a longitudinal quasi-experimental design was used to evaluate the impact of the GBPS pilot after full implementation, and in contrast to the baseline CVD medical expenditure [20]. We additionally adopted a segmented autoregressive integrated moving average (ARIMA) model to estimate the change as a result of the type of medical expenditure, discharge number, and medical expenditure per capita. After controlling for the baseline trend and other covariates, the ITS models were used to evaluate the impact on the expenditure after the GBPS pilot had been fully implemented, by instant change and trend change. A total of 48 monthly time points from January 2009 to December 2012 were collected, and the interrupt points were set at April 2010 and April 2011. The segmented regression model with two intervention points was built as the following equation:

Yt = β_0_ + β_1_ × time_t_ + β_2_·inter1 + β_3_ × trend change1_t_ + β_4_ × inter2 + β_5_ × trend change2_t_ + β_6_ × male percent + β_7_ × patient elder percent + β_8_ × quarter2 + β_9_ × quarter3 + β_10_ × quarter4 (1)

In this formula, to represent the linear time trend, time was a continuous variable. Inter1 and inter2 were indicators for time t occurring before or after the GBPS pilot in 2010 and full implementation in 2011. Before the GBPS pilot, inter1 and inter2 were 0. After the GBPS pilot, inter1 changed to 1. After the GBPS full implementation, inter2 changed to 1. Trend change1 and trend change2 represent continuous variables, counting the number of months at time t from the GBPS pilot and full implementation and to reflect the monthly trend change after interrupt points. The male percentage and percentage of patients older than 65 were adopted to reflect the covariate by gender and age. The quarter2, quarter3, and quarter4 acted as dummy variables to divide the quarters’ impact. All outcome indicators were substituted into the above ITS model and focused on the GBPS instant change and trend change. To avoid uncertainty in the GBPS’ impact, we conducted an uncertainty analysis by placing the monthly data of the same outcomes of respiratory diseases (ICD10: J00-J99) into the ITS model from the same time period.

STATA 15.0 was used to perform all analysis. The Dickey–Fuller test proved the data to be stable during the time series. Based on the autocorrelation function and partial autocorrelation function plots of independent variables, the data proved to be fit with AR (Auto Regressive) and MA (Moving Average) terms. The Ljung-Box Q statistic was used to evaluate the null hypothesis and assess if the residuals were white noise, which indicates the ARIMA model is well fit to the data [21].

## 3. Results

### 3.1. Descriptive Trends

The annual medical expenditure and discharge number are shown in Table 1. Over the four-year period, the annual medical expenditure grew continuously, with 26.8% total growth. However, the rate of increase in 2010 (14.4%) was much higher than that of following two years (5.3% on average). The discharge number and expenditure per capita in the four years show a similar trend. The drug expenditure increased by 24.2%, although the share of drug expenditure as a part of the total expenditure experienced a decreasing trend. In 2011, the share of drug expenditure dropped three percent as compared to its share in 2009. Inversely, the share of diagnosis expenditure and cure expenditure continued to increase, with total increase rates of 19.3% and 10.0%, respectively.

**Figure 1:**
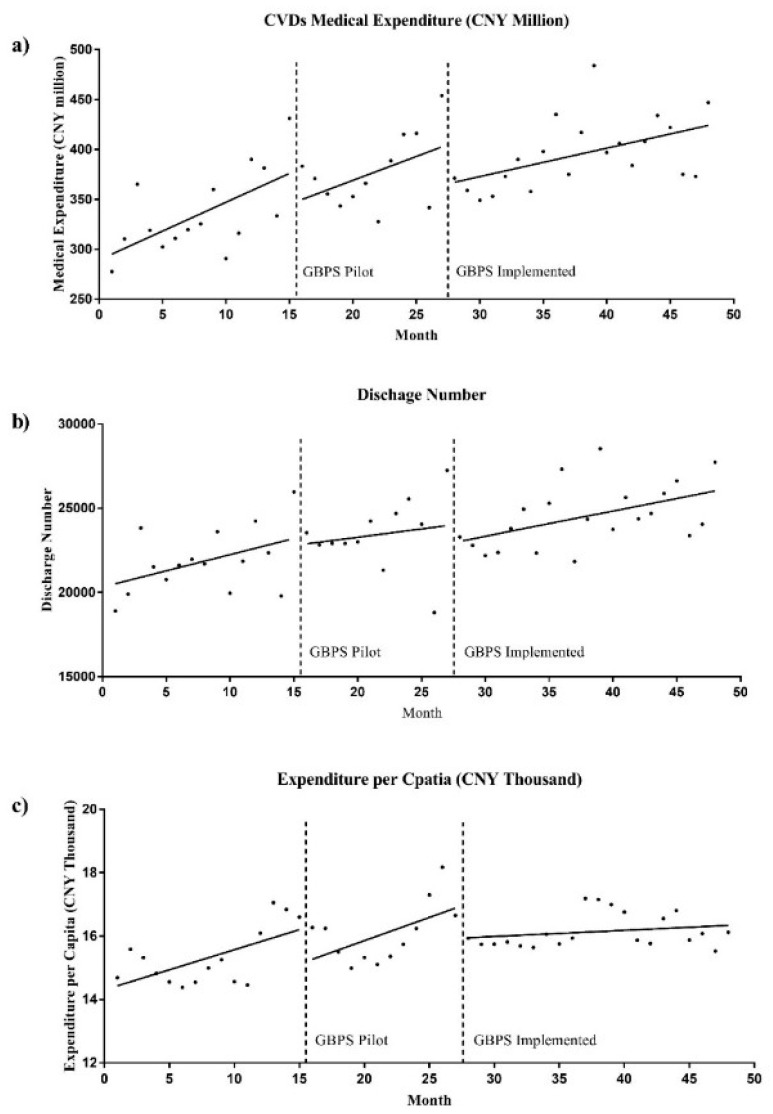
Monthly trend of medical expenditure, discharge number and expenditure per capita in study period.

**Figure 2:**
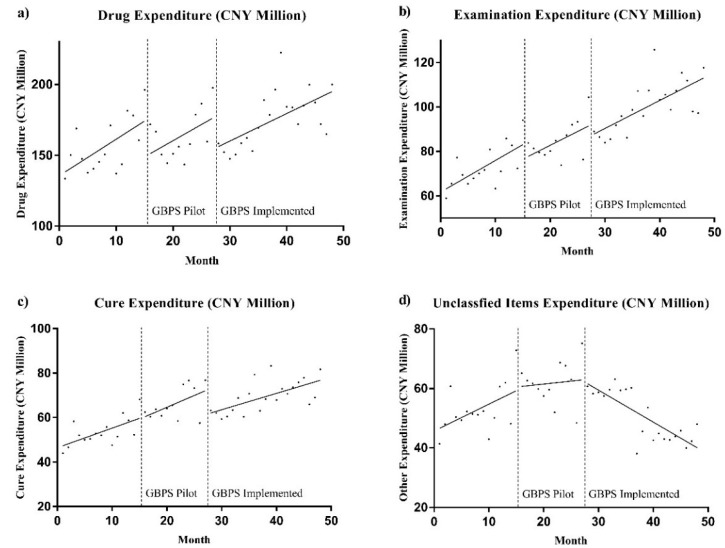
Monthly trend of different types of medical expenditure in study period.

Figure 1 and Figure 2 display the monthly trend of medical expenditure and discharge number. The line found on each scatter diagram represents the regression curve for each outcome indicator and the three study time segments. After the GBPS full implementation, the monthly trend of medical expenditure and expenditure per capita slowed down—in contrast to pre-launch—with noticeable breakpoints (Figure 1a,c). However, the trends of discharge number in the three segments experienced no significant change, although the breakpoint between the GBPS pilot and the GBPS full implementation still exists (Figure 1b).

Figure 2 reflects the monthly trend of the different types of medical expenditure during the study period. Drug expenditure demonstrated the greatest reaction to the GBPS, with breakpoints after the GBPS pilot and the GBPS full implementation (Figure 2a). The examination and cure expenditures were influenced minimally by the GBPS (Figure 2b,c). Finally, the GBPS had an immediate impact on unclassified expenditure, and continued to do so over the long-term (Figure 2d).

### 3.2. Interrupted Time Series Analysis

Table 2 displays the ITS results for medical expenditure, discharge number, and expenditure per capita. After the GBPS full implementation, the medical expenditure dropped abruptly by CNY 55.71 million (*p* < 0.001), which represented 12.3% of the medical expenditure in the month prior to the GBPS implementation. Moreover, the monthly increasing speed of medical expenditure decreased by 70.4%, or CNY 4.23 million after the GBPS full implementation (*p* = 0.011). 

The discharge number of CVDs experienced a similar change. The instant decrease of the discharge number was 10.4% of the number in the month prior to the GBPS full implementation (*p* < 0.001). Additionally, the monthly trend decrease of discharge number was 225.55 (*p* = 0.021), higher than the baseline increasing speed of 165.69 (*p* = 0.001). However, the GBPS pilot and full implementation had no significant impact on the expenditure per capita.

The drug and cure expenditures were influenced greatest by the GBPS. Table 3 displays the results of the GBPS’ impact on different types of medical expenditure by ITS. From Table 3, the GBPS pilot only had significant impact on the drug expenditure, which dropped abruptly by 9.3% and demonstrated a decrease of CNY 1.26 million (*p* = 0.003). After the GBPS full implementation, the drug expenditure dropped further by 14.4% (*p* < 0.001). With respect to the cure expenditure, the expenditure dropped by 21.2% (*p* < 0.001), and the increasing trend inversed, decreasing by CNY 1.16 million per month (*p* < 0.001). Finally, with respect to the examination and unclassified items expenditures, the GBPS had no significant impact in the short-term. In the long-term, however, the monthly growth trend decreased by CNY 1.55 million (*p* = 0.013) and CNY 2.41 million (*p* < 0.001), respectively.

### 3.3 The Sensitivity Analysis

We applied the ITS model to test the stability of GBPS’ impact on the monthly medical expenditure of inpatients with respiratory disease. This test showed similar results. After the GBPS pilot and full implementation, the medical expenditure and discharge number of respiratory disease experienced a significant drop, the monthly trend slowed, and the expenditure per capita experienced no significant impact (Table A1). For different types of expenditure of respiratory disease, the drug and cure expenditures also noticeably decreased after the GBPS pilot and full implementation in the short-term. The expenditure of unclassified items, such as surgery and transfusion, experienced the greatest monthly drop, in contrast to the other three types of expenditure (Table A2).

## 4. Discussion

Our results confirmed the effectiveness of the GBPS in controlling expenditure, at least in association with CVDs. Based on the results of previous studies, we further demonstrate that the GBPS does not only reduce expenditure in the short-term but also slows the increasing rate of expenditure growth in the long-term. From the results, there exists a significant trend decrease after the GBPS full implementation. Consequently, we considered the GBPS in Shanghai to be a powerful policy for controlling the expenditure of CVDs. This finding had great actual meaning for Shanghai was one of the earliest places to launch the GBPS in China. In that case, the impact of the GBPS in Shanghai provides reference to other provinces in China which launched the GBPS recent years [22]. Recently, the Chinese government tried mixed payment system launch, such as diagnosis related groups (DRGs), which were also on the base of the GBPS [23,24].

Earlier in this paper, we referred to a study that described the relationship between the GBPS and changes in physicians’ prescribing behavior for some diseases [25]. While there is a cause–effect relationship, other factors also need to change to control for rising expenditure [26]. For example, in this study, the ITS results show that the decline of medical expenditure after the GBPS implementation was mainly caused by the reduction in patient discharges. The trend chart shows that the discharge number reached its height several months prior to the GBPS launch, likely because hospitals wanted to avoid higher medical expenditure at the end of year. In the immediate months post-GBPS implementation, the discharge number decreased significantly, as hospitals may have already shifted inpatients with higher medical expenditures to other hospitals [27]. Prior research on the GBPS’ impact in rural China also verified the phenomenon of physicians not recommending patients for hospitalization when they need it to work within the budget [28]. Moreover, our results showed the GBPS had no significant influence on medical expenditure per capita. This also indirectly supports the conclusion that the GBPS controls expenditure by reducing patient numbers.

Where the GBPS can negatively impact a hospital’s budget, hospitalization options may not be offered to patients who need inpatient services. If the annual budget is set too low, the GBPS can negatively impact medical service quality and patient experience [29]. While there are studies on the GBPS’ impact on quality in limited settings (e.g., impact by disease), further long-range research is needed to assess the impact of annual budgets on service quality across multiple diseases [30,31]. It may also be beneficial to assess the impact of combining a prospective payment system with FFS, and payment by DRGs for specific diseases [32,33]. 

The tertiary hospitals showed a stronger reaction to the GBPS, in contrast to the secondary hospitals. Additionally, medical expenditure dropped sharply after the GBPS was fully implemented, whereas the GBPS pilot showed no statistically significant changes. Previous research also showed that the GBPS had a more prominent impact in changing physician behavior in larger general hospitals [32,34]. This may be because tertiary hospitals have more methods to control for expenditure, such as shifting inpatients to secondary and primary hospitals. Tertiary hospitals in Shanghai make up 70.87% of the total medical expenditure among healthcare institutions. Based on the theory of expected income, they consequently have a greater ability to undertake loss associated with the GBPS [34,35].

The drug and cure expenditures were influenced most by the GBPS. With respect to the drug expenditure, the impact of the GBPS was visible even during the GBPS pilot. In contrast, previous related studies had the inverse results. These studies showed that physicians increased the drug expenditure under the GBPS to recover for the loss [36,37]. However, because the drug expenditure was the most important part of the total expenditure in Shanghai, the implementation of the GBPS in Shanghai focused on setting a budget for drug expenditure. This measure controlled the drug expenditure immediately and effectively. And, as secondary hospitals in China rely more heavily on drug expenditure, this expenditure became controlled after the GBPS pilot [35]. The reason for this control was mainly because physicians switched the original brand prescription drugs to less expensive generic drugs [21]. 

Under the price regulation policy for medical services in China, a significant portion of physicians’ income originates from the examination expenditure [38]. Consequently, physicians are potentially less likely to reduce the number of examination services in contrast to, for example, treatment-related services [39,40,41], This may be a reason why the GBPS influenced the examination expenditure more than it influenced the cure expenditure.

This study has a few limitations related to access to targeted information and time. First, because we collected the daily medical expenditure and discharge number by the sum of all hospitalizations insured by UEBMI in Shanghai, we could not distinguish the outcomes and variables between different hospitals. However, this data is not accessible to the general public, and we consequently only assessed the effect among hospitals by the GBPS pilot and GBPS full implementation. Second, as the GBPS sets a budget—to be paid by UEBMI—for total medical expenditure, and not just for CVDs, our results could not reflect the impact of the GBPS in Shanghai beyond what is associated with the treatment of CVDs. A budget by disease does not exist. Nonetheless, CVDs represent a tremendous contribution to the total expenditure and discharge number, and our sensitivity analysis showed that the GBPS has a similar impact on other diseases, but with varying degrees of influence. Third, the study period was limited in four years (48 months) as a result of potential policy interference. A longer time period could have potentially provided more stability to the results.

## 5. Conclusions

Finally, in consideration of the study’s scope and understanding the limitations, our two main conclusions are: First, the GBPS is an effective solution to control for the expenditure of inpatients diagnosed with CVDs, both in the short and long-terms. This effect of controlling was mainly by the decrease of discharge number instead of reducing the burden of inpatients. Second, we concluded that the drug and cure expenditure were influenced the most by the GBPS, in comparison to other types of expenditure. The next step is to study the impact of the GBPS on other diseases.

## Figures and Tables

**Table 1:** Basic information of the sample during study period.

	2009	2010	2011	2012
**General Condition**				
Medical Expenditure (Million CNY)	3887.58	4449.21	4601.65	4929.96
Discharge Number	259,903	279,166	284,466	300,821
Gender (Male, %)	49.5	49.6	49.7	50.3
Age (>65, %)	72.6	72.6	71.4	69.3
Expenditure per Capita (Thousand CNY)	14.96	15.94	16.18	16.39
**Expenditure Pattern, million CNY (%)**				
Drug	1807.75 (46.5)	1955.65 (44.0)	1984.57 (43.1)	2246.06 (45.6)
Diagnosis and Examination	846.68 (21.8)	990.04 (22.3)	1098.53 (23.9)	1283.99 (26.0)
Cure	622.80 (16.0)	766.05 (17.2)	795.11 (17.3)	870.11 (17.6)
Unclassified items	610.36 (15.7)	737.47 (16.6)	723.43 (15.7)	529.80 (10.7)

**Table 2:** Segmented autoregressive integrated moving average analysis of medical expenditure.

	Total Medical Expenditure (Million CNY)			Discharge Number			Expenditure per Capita (Thousand CNY)		
	β ^a^	SE ^b^	*p* Value	β ^a^	SE ^b^	*p* Value	β ^a^	SE ^b^	*p* Value
Baseline trend ^c^	6.01	0.92	0.000	165.69	49.57	0.001	0.00	0.06	0.992
Level change 1 ^d^	−18.92	11.99	0.115	−50.87	673.73	0.940	−0.24	0.60	0.686
Trend change 1 ^e^	−2.78	1.50	0.064	−64.46	56.41	0.253	−0.00	0.07	0.978
Level change 2 ^f^	−55.71	14.48	0.000	−2845.89	814.57	0.000	0.48	0.36	0.182
Trend change 2 ^g^	−4.23	1.67	0.011	−225.55	97.74	0.021	0.05	0.05	0.327
Gender (Male, %)	−1000.62	1140.97	0.380	−75,617.83	53,998.75	0.161	30.54	26.50	0.249
Age (>65, %)	−2778.84	603.75	0.000	−183,303	39737	0.000	40.25	15.00	0.007
second quarter	−61.14	12.85	0.000	−2282.78	596.46	0.000	−0.19	0.31	0.540
third quarter	−103.91	15.36	0.000	−4576.09	946.30	0.000	0.57	0.41	0.167
fourth quarter	−96.21	14.57	0.000	−4162.28	983.15	0.000	0.98	0.36	0.006
Cons	2861.98	741.88	0.000	193,796.5	45,640.79	0.000	−44.52	19.07	0.020
AR1	−0.29	0.26	0.260	−0.46	0.30	0.127	0.23	0.25	0.352
AR2	−0.67	0.15	0.000	−0.63	0.18	0.000	−0.60	0.14	0.000
AR3	−0.39	0.24	0.097	−0.44	0.22	0.047	/	/	/
Ljung−Box Q	21.13	/	0.341	33.19	/	0.059	24.34	/	0.277

^a^ Coefficient of each variety; ^b^ Standard error of each variety; ^c^ Baseline time trend of dependent variable; ^d, e, f, g^ Instant change and time trend change of GBPS pilot and implemented.

**Table 3:** Segmented autoregressive integrated moving average analysis of medical expenditure (Million CNY).

	Drug			Examination			Cure			Unclassified Items		
	β ^a^	SE ^b^	*p* Value	β ^a^	SE ^b^	*p* Value	β ^a^	SE ^b^	*p* Value	β ^a^	SE ^b^	*p* Value
Baseline trend ^c^	2.66	0.47	0.000	0.34	0.38	0.370	0.91	0.24	0.000	0.86	0.37	0.020
Level change 1 ^d^	−18.26	6.62	0.006	−3.54	4.41	0.423	1.24	3.25	0.703	2.89	4.88	0.553
Trend change 1 ^e^	−1.26	0.43	0.003	−0.22	0.39	0.577	0.06	0.29	0.824	−0.93	0.67	0.168
Level change 2 ^f^	−28.31	6.67	0.000	−6.26	5.68	0.271	−16.28	2.07	0.000	−5.68	5.51	0.302
Trend change 2 ^g^	−0.48	0.89	0.587	−1.55	0.62	0.013	−1.16	0.31	0.000	−2.41	0.67	0.000
Gender (Male, %)	−1033.10	590.34	0.080	318.55	303.95	0.002	−15.62	223.36	0.944	−57.80	234.29	0.805
Age (>65, %)	−1165.90	299.06	0.000	−694.85	226.18	0.295	−568.79	96.63	0.000	−773.9	95.30	0.000
second quarter	−30.99	6.39	0.000	−17.05	2.83	0.000	−10.11	1.92	0.000	−10.38	3.24	0.001
third quarter	−52.78	6.61	0.000	−18.14	4.81	0.000	−16.79	2.35	0.000	−18.18	3.80	0.000
fourth quarter	−52.89	8.28	0.000	−15.0	4.50	0.001	−14.24	2.30	0.000	−14.86	2.93	0.000
Cons	1524.78	394.11	0.000	357.62	286.07	0.182	476.83	147.70	0.001	647.35	170.19	0.000
AR1	0.41	0.22	0.064	−0.60	0.21	0.004	−0.02	0.12	0.861	0.31	0.21	0.131
AR2	−0.62	0.15	0.000	−0.45	−0.19	0.020	−0.64	0.16	0.000	/	/	/
MA1	−1.00	0.26	0.000	/	/	/	/	/	/	/	/	/
Ljung−Box Q	23.31	/	0.384	31.15	/	0.071	29.69	/	0.126	24.23	/	0.335

^a^ Coefficient of each variety; ^b^ Standard error of each variety; ^c^ Baseline time trend of dependent variable; ^d, e, f, g^ Instant change and time trend change of GBPS pilot and implemented.

## Appendix A

Appendix A shows the result of sensitivity analysis, which used the monthly expenditure of respiratory diseases (ICD10: J00-J99) in Shanghai during the same time period. We used the same time series model to evaluate the instant change and trend change of GBPS piloted and implemented. The outcome indicators included the medical expenditure (total, drug, examination, cure, and other items), discharge number, and expenditure per capita. Table A1 shows the results of total medical expenditure, discharge number and expenditure per capita of ARIMA, and Table A2 shows the results of expenditure of drugs, examination, cure, and unclassified items of ARIMA. The results of sensitivity analysis show the GBPS also dropped the total medical expenditure and discharge number of inpatients with respiratory diseases in short and long term, and the expenditure of drug and cure were more sensitive to the GBPS.

**Table A1:** Segmented autoregressive integrated moving average analysis of medical expenditure of respiratory disease.

	Total Medical Expenditure (Million CNY)			Discharge Number			Expenditure per Capita (Thousand CNY)		
	β ^a^	SE ^b^	*p* Value	β ^a^	SE ^b^	*p* Value	β ^a^	SE ^b^	*p* Value
Baseline trend ^c^	2.40 ***	0.44	0.000	187.87	56.23	0.001	−0.01	0.03	0.803
Level change 1 ^d^	−19.83 **	5.77	0.001	−1933.02	716.52	0.007	−0.05	0.30	0.867
Trend change 1 ^e^	−1.07 *	0.45	0.017	−67.09	54.51	0.218	0.02	0.02	0.405
Level change 2 ^f^	−14.31 **	5.24	0.006	−1798.85	565.95	0.001	0.05	0.20	0.800
Trend change 2 ^g^	0.17	0.46	0.711	13.97	50.94	0.784	−0.02	0.02	0.240
Gender (Male, %)	−252.26	136.86	0.065	−38,323.15	15,388.11	0.013	−6.12	5.31	0.249
Age (>65, %)	197.38 ***	63.37	0.002	20,786.77	7752.75	0.007	−2.51	2.89	0.386
second quarter	−7.19	4.07	0.077	−470.57	478.06	0.325	−0.12	0.17	0.479
third quarter	−9.90 *	4.45	0.026	−645.27	581.55	0.267	−0.20	0.23	0.373
fourth quarter	−10.63	5.48	0.053	−889.35	669.29	0.184	−0.03	0.19	0.890
Cons	91.55	97.16	0.346	17,301.38	10,469.47	0.198	5.40	4.05	0.182
AR1	−0.10	0.14	0.475	−0.13	0.17	0.444	−0.59 ***	0.15	0.000
AR2	−0.68 ***	0.12	0.000	−0.65	0.12	0.000	−0.56 **	0.19	0.004
Ljung−Box Q	10.73	/	0.979	15.23	/	0.852	12.58	/	0.923

^a^ Coefficient of each variety; ^b^ Standard error of each variety; ^c^ Baseline time trend of dependent variable; ^d, e, f, g^ Instant change and time trend change of GBPS pilot and implemented.

**Table A2:** Segmented autoregressive integrated moving average analysis of medical expenditure of respiratory disease (Million CNY).

	Drug			Examination			Cure			Unclassified Items		
	β ^a^	SE ^b^	*p* Value	β ^a^	SE ^b^	*p* Value	β ^a^	SE ^b^	*p* Value	β ^a^	SE ^b^	*p* Value
Baseline trend ^c^	1.43	0.25	0.000	0.61	0.14	0.000	0.22	0.05	0.000	0.16	0.03	0.000
Level change 1^d^	−13.39	3.34	0.000	−3.95	1.88	0.036	−1.92	0.50	0.000	−0.74	0.49	0.128
Trend change 1^e^	−0.78	0.28	0.005	−0.17	0.14	0.248	−0.06	0.04	0.125	−0.09	0.04	0.021
Level change 2^f^	−9.22	3.17	0.004	−3.25	1.58	0.040	−1.83	0.52	0.000	−0.02	0.41	0.965
Trend change 2^g^	0.30	0.29	0.296	0.03	0.13	0.836	−0.06	0.04	0.118	−0.09	0.04	0.015
Gender (Male, %)	−147.47	79.00	0.062	−77.60	44.29	0.080	−19.38	14.48	0.181	−6.76	10.07	0.502
Age (>65, %)	134.56	36.30	0.000	53.79	19.95	0.007	20.01	5.31	0.000	−3.41	7.11	0.631
second quarter	−5.29	2.45	0..031	−1.28	1.22	0.29	−0.19	0.35	0.589	−0.01	0.36	0.987
third quarter	−6.49	2.50	0.009	−1.63	1.48	0.269	−0.45	0.38	0.267	−0.73	0.48	0.125
fourth quarter	−6.21	3.08	0.043	−2.48	1.77	0.161	−0.50	0.49	0.308	−0.79	0.53	0.137
Cons	41.76	55.88	0.455	26.39	30.02	0.379	5.29	10.23	0.605	11.97	8.06	0.138
AR1	−0.06	0.12	0.617	−0.08	0.19	0.668	−0.23	0.19	0.233	−0.41	0.19	0.027
AR2	−0.68	0.13	0.000	−0.64	0.13	0.000	−0.67	0.12	0.000	−0.56	0.17	0.001
Ljung−Box Q	11.11	/	0.973	14.45	/	0.885	15.50	/	0.840	29.03	/	0.144

^a^ Coefficient of each variety; ^b^ Standard error of each variety; ^c^ Baseline time trend of dependent variable; ^d, e, f, g^ Instant change and time trend change of GBPS pilot and implemented.
